# Sialic acids modulate immune responses in cancer: Therapeutic opportunities

**DOI:** 10.1016/j.jbc.2026.111245

**Published:** 2026-02-05

**Authors:** Eleanor E. Bashian, James C. Paulson, Peng Wu

**Affiliations:** 1 Department of Molecular and Cellular Biology, The Scripps Research Institute, La Jolla, California, USA; 2 Department of Immunology and Microbiology, The Scripps Research Institute, La Jolla, California, USA

**Keywords:** sialic acid, cancer immunotherapy, Siglecs, Selectins, glycoimmune, checkpoint, hypersialylation, Immune evasion, glycan-targeted therapies

## Abstract

The development of therapies that boost antitumor immunity has transformed cancer treatment. While the efficacy of traditional therapies, such as chemotherapy and radiation therapy, is limited by toxicity and resistance, forms of immunotherapy, including immune checkpoint blockade therapies and engineered cellular therapies, have shown unprecedented success for certain patient populations. Despite these advances, therapeutic resistance remains a significant barrier, and alternative therapies are needed to overcome immune evasion mechanisms. One prominent evasive mechanism utilized by tumor cells is hypersialylation, the overexpression of glycans capped with sialic acid on the cell surface. This review focuses on the immunosuppressive role of sialic acid in cancer and highlights opportunities to target sialic acid and its binding proteins, offering a promising therapeutic perspective to counteract resistance and improve patient outcomes.

Despite major advances in certain immunotherapy approaches, their limited efficacy in patients with primary or adaptive resistance highlights the need for therapies that target alternative inhibitory pathways. Glycosylation is a post-translational modification that contributes to the diversity of proteins and lipids and makes up the glycocalyx on the surface of cells. On healthy cells, the glycocalyx plays an essential role in normal cellular functions, including cell-cell interactions, trafficking, and signal transduction. In cancer, these processes are dysregulated by aberrant glycosylation. One particularly prominent form of tumor-associated glycosylation is hypersialylation, the upregulation of glycans capped with sialic acid (1). Sialic acid is thought to function as a self-associated molecular pattern to suppress immune responses, allowing cancers with hypersialylation to evade immune attack (2).

Here, we review the therapeutic vulnerabilities of hypersialylated cancers, as well as recent strategies to remodel the tumor sialoglycome and associated interactions. We discuss how sialic acid dampens anti-tumor immunity and highlight suppressive interactions with sialic acid-binding proteins. We examine clinical therapies targeting two families of sialic acid receptors: sialic acid-binding immunoglobulin-like lectins (Siglecs) and Selectins, and we briefly discuss the progress of novel therapies in relevant preclinical models. These efforts reflect growing interest in glycan-targeted therapies that hold tremendous potential to enhance or synergize with current immunotherapy approaches.

## Sialic acid is a marker of “self” exploited by cancer

### Hypersialylation as a hallmark of cancer

**Figure 1:**
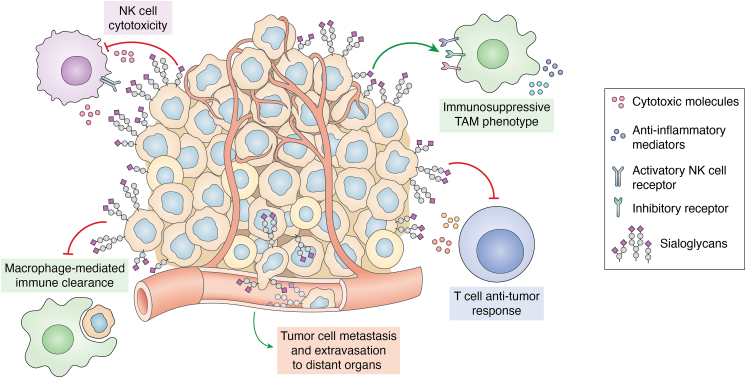
**Hypersialylation is a hallmark of cancer.** Sialic acid decorating tumor cells contributes to cancer progression by suppressing anti-tumor immunity and promoting cancer spread. Immunosuppressive sialoglycans dampen T cell function in the tumor microenvironment (TME), prevent phagocytosis of cancer cells by macrophages, inhibit NK cell cytotoxicity, and promote an immunosuppressive tumor-associated macrophage (TAM) phenotype. Tumor cell-associated sialic acid also facilitates tumor cell metastasis to distant sites. *Red flat arrows* indicate inhibition and *green arrows* indicate activation.

As the terminal sugar on N- and O-glycans and glycolipids, sialic acid plays a crucial role in marking cells as “self” to the immune system to prevent unwanted immune responses. A hallmark of cancer is hypersialylation, characterized by the upregulation of glycans capped by sialic acids, which contribute to cancer progression by promoting immune evasion, metastasis, and angiogenesis (Fig. 1) (1, 3). Hypersialylation masks tumor antigens and dampens the anti-tumor immune response (1, 4, 5). Increased sialylation also contributes to the spread of cancer to distant organs by enhancing tumor cell migration into tissues and promoting the formation of new blood vessels providing nutrients and oxygen to support tumor growth (6, 7, 8, 9, 10). Although aberrant sialylation has long been recognized in cancer, its therapeutic exploitation is still an emerging focus, and clinically standardized assays remain limited to select antigen-specific assays (*e.g.* CA 19–9 for pancreatic cancer), underscoring the need for development of robust biomarkers to guide patient selection (11, 12).

Hypersialylation of cancer cells largely results from overexpression of sialyltransferases. There are 20 sialyltransferases that add terminal sialic acids in different linkages to N-linked and O-linked glycans, as well as glycolipids. Upregulation of sialyltransferases is a common feature of many types of cancers and often holds prognostic value (13, 14, 15). For example, high expression of ST3Gal6 in multiple myeloma is correlated with shorter overall survival compared to patients with low expression (16). Multiple reports have indicated upregulation of ST6Gal1 in ovarian cancer, pancreatic ductal adenocarcinoma, and breast cancer (17, 18, 19, 20). In ovarian cancer, high expression of ST6Gal1 was associated with more aggressive disease features and shorter survival (20). Hypersialylation has also been linked to resistance to chemotherapy, chemoradiation, and immunotherapy (21, 22, 23, 24, 25).

### Sialic acid as ligands of Siglecs that modulate immune responses

**Figure 2:**
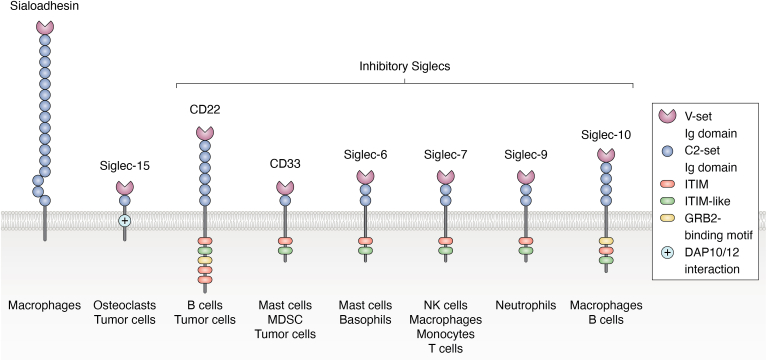
**Siglecs regulate immune cell activity and play key roles in antitumor immunity**. Siglecs are composed of a V-set binding domain and variable numbers of C2-set Ig-like domains. Most Siglecs function as inhibitory receptors, modulating immune responses through immunoreceptor tyrosine-based inhibitory motifs (ITIMs) or ITIM-like motifs in their cytoplasmic domains, while a few are activatory and interact with immunoreceptor tyrosine-based activatory motif (ITAM) adapter proteins through a positively charged amino acid in their transmembrane domain. Siglecs are primarily expressed on immune cells, though expression on tumor cells has been documented for some. Adapted from Crocker *et al.*, Nat Rev Immunol (**2007**), which describes the complete Siglec family.

One way in which sialic acid-containing glycans (sialoglycans) can dampen anti-tumor immunity is through their interactions with sialic acid-binding immunoglobulin-like lectins (Siglecs) that negatively regulate immune cell signaling. Siglecs are a diverse family of receptors differentially expressed on white blood cells that comprise the innate and adaptive immune system. The family is made of conserved members (*i.e.* Siglec-1 (CD169), Siglec-2 (CD22), Siglec-4 (MAG), and Siglec-15) that share high homology between species and variable Siglec-3 (CD33)-related members that diverged between species (Fig. 2). Most CD33-related members in humans have functional orthologs in mice (*i.e.* Siglec-8/Siglec-F, Siglec-10/Siglec-G) (26). Structurally, each Siglec contains a V-set sialic acid-binding domain that mediates binding to sialoglycan ligands, and a variable number (1–16) of C2-set Ig-like domains in its extracellular domains (Fig. 2) (27).

Siglecs are generally regarded as signaling co-receptors; however, most Siglecs are also endocytic receptors that either constitutively cycle between the cell surface and endosomes or are induced to endocytose upon ligation or cross-linking (28). CD169 is the only Siglec without intracellular signaling domains and is thought to primarily function as an endocytic receptor (29). The majority of the Siglecs are inhibitory co-receptors (*e.g.* CD22, CD33, Siglec-5-12) due to immunoreceptor tyrosine-based inhibitory motifs (ITIMs) in their cytoplasmic domains that, when phosphorylated, recruit SH2 domain-containing protein tyrosine phosphatases (SHP1 and SHP2) to negatively regulate immune responses (27). A few Siglecs (MAG, Siglec-15, Siglec-14/16) are activatory and contain positively charged amino acids in their transmembrane regions that mediate interaction with immunoreceptor tyrosine-based activation motif (ITAM) adaptor DAP12 (28). These signaling domains with opposing functions allow Siglecs to dynamically regulate the immune responses in a highly context-specific manner.

Siglecs interact with sialic acid ligands with distinct but overlapping ligand specificity. Ligand specificity depends on the linkage of sialic acid to the penultimate monosaccharide in an α2-3, α2-6, or α2-8 connection, as well as the underlying glycan scaffold (27, 30, 31, 32). For example, CD22 recognizes sialic acid in the NeuAcα2-6Galβ1-4GlcNAc epitope as a preferred glycan scaffold, while Siglec-7 preferentially recognizes di-sialylated epitopes, such as NeuAcα2-3Galβ1-3(NeuAcα2-6)GalNAc in an O-linked glycan scaffold or NeuAcα2-8NeuAcα2-3Galβ1-3GalNAc in a glycolipid scaffold (33). Sulfation of sialylated glycans is also a critical element of Siglec ligand specificity (32, 34, 35, 36). Siglec-8 and its murine paralog Siglec-F strongly prefer ligands with sulfation on the penultimate galactose (NeuAcα2-3[6S]Galβ1-4GlcNAc) (37, 38, 39). Moreover, for the aforementioned CD22 and Siglec-7, 6-sulfation on the GlcNAc or GalNAc of their respective ligands strongly increases avidity (34, 40, 41). The glycoprotein carrying the sialylated glycans can also play a critical role in ligand recognition. Using Siglec-7 as an example, the mucin-type glycoprotein CD43 was found to be a preferred ligand for Siglec-7 on human leukemia cells (42). Even the spatial distribution of O-linked glycans on mucin type glycoproteins can have a profound impact on the binding of Siglec-7 and other Siglecs (42, 43, 44, 45). In summary, it is now clear that ligand recognition by Siglecs is strongly context dependent. For a Siglec on one cell recognizing glycoprotein ligands on another cell, many factors come into play as to which glycoproteins serve as the predominant ligands in that context.

### Sialic acid as ligands of selectins mediating cancer metastasis and immune cell trafficking

**Figure 3:**
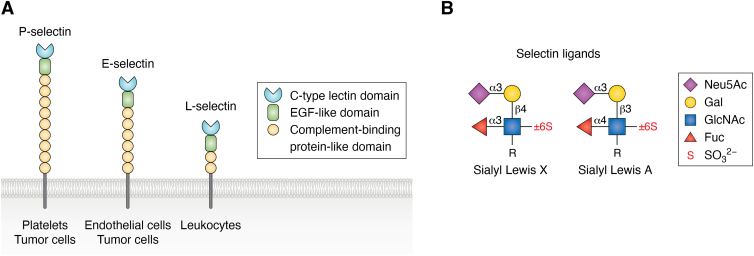
**Selectins mediate leukocyte trafficking and promote cancer spread**. *A*, depiction of the three members of the selectin family, named for their predominant expression. Each selectin has a Ca2+-dependent carbohydrate binding domain, an epidermal growth factor (EGF)-like domain, and variable complement-binding protein-like domains. Selectins bind to the tetrasaccharide Sialyl Lewis X (SLex; NeuAcα2-3Galβ1-4(Fucα1-3)GlcNAc), or its isomer Sialyl Lewis A (SLea; NeuAcα2-3Galβ1-3(Fucα1-4)GlcNAc), displayed on protein carriers (*B*). In the cancer setting, selectin ligands on tumor cells facilitate metastasis through interactions with endothelial cells and platelets.

Sialoglycans can also contribute to cancer metastasis as ligands for the selectin family of glycan receptors known for their roles in leukocyte trafficking. Selectins are a family of three closely related proteins named according to their expression on endothelial cells (E-selectin), leukocytes (L-selectin), and platelets and endothelial cells (P-selectin) (25). Structurally, selectins share an extracellular region composed of a Ca^2+^-dependent lectin-like domain, and epidermal growth factor-like domain, and a series of 2 to 9 complement-binding protein-like domains followed by a short cytoplasmic tail (Fig. 3) (15, 46). The minimal recognition motif for all three selectins is the tetrasaccharide Sialyl Lewis X (SLe^x^; NeuAcα2-3Galβ1-4(Fucα1-3)GlcNAc) and its isomer Sialyl Lewis A (SLe^a^; NeuAcα2-3Galβ1-3(Fucα1-4)GlcNAc), which can be displayed as a terminal sequence on N-linked and/or O-linked glycans of glycoproteins and glycolipids (46). However, L-selectin and P-selectin exhibit specificities distinct from each of the other two. L-selectin recognizes the sulfated-SLe^x^ (*e.g.* NeuAcα2-3Galβ1-4(Fucα1-3)[6S]GlcNAc) expressed on glycoproteins of endothelial cells in lymphoid organs. P-selectin exhibits strong preference for SLe^x^ on O-glycans on P-selectin glycoprotein ligand-1 (PSGL-1) that displays sulfated tyrosine a few amino acids away from the glycan (46, 47, 48). The specificities of the selectin-mediated interactions with their ligands provide different contexts for homing and trafficking of leukocytes to inflamed tissues and lymphoid organs, where the selectins play the role of pulling the cells out of the flow of blood and slowing them down through a process known as “rolling and tethering” prior to their adhesion and transmigration into the tissues (49, 50, 51, 52). As will be discussed below, in the context of cancer, some cancers express selectin ligands, which can result in selectin-mediated metastasis to remote sites if the cancer cells are released into the blood, contributing to disease progression (15).

## Reducing cancer sialylation enhances immune responses

The suppressive nature of hypersialylation has been recognized for decades. As early as the 1980s, the “neuraminidase effect” was established, demonstrating that removing sialic acid from the surface of immune cells enhances activation (5). Recent work has explored the therapeutic potential of removing sialic acid using more targeted strategies, documenting the effect of reducing hypersialylation on cancer progression (53, 54, 55, 56). The sections below review genetic, pharmacological, and enzymatic strategies that are being evaluated to reprogram the tumor microenvironment to enhance antitumor immunity.

### Engineering cancer cells deficient in sialylation

One approach to understanding the importance of hypersialylation in cancer progression has been to genetically engineer cancer cells with reduced sialylation. Ablation of the enzyme cytidine monophosphate N-acetylneuraminic acid synthetase (CMAS) that is responsible for the sialyltransferase donor substrate CMP-NeuAc shuts down sialylation entirely. Knocking out the *Cmas* gene dramatically reduced tumor burden in a murine model of metastatic breast cancer (57).Interestingly, however, knocking out CMAS in the MC38 murine colorectal cancer model promoted tumor growth, indicating that the impact of tumor cell sialylation could be context dependent (58). Another critical enzyme in sialoglycan biosynthesis is Slc35a1, the transporter required to move sialic acid into the Golgi for subsequent transfer to glycans. Knocking out Slc35a1 in a murine model of melanoma reduced tumor progression and enhanced survival (59). Several groups have used a more selective strategy to understand importance of different linkages of sialic acid by knocking out individual sialyltransferases. GC *et al.* uncovered a protumorigenic role of ST6Gal1 in glioblastoma and demonstrated that knockdown of the enzyme resulted in prolonged survival in a murine model (60). Similarly, knockdown of ST3Gal6, an enzyme that plays a key role in selectin ligand biosynthesis, prolonged survival in a murine model of multiple myeloma (16).

### Inhibiting sialyltransferases pharmacologically

Several strategies have been explored to reduce cancer cell sialylation pharmacologically using inhibitors of sialyltransferases. One pan-sialyltransferase inhibitor is peracetylated 3F_ax_Neu5Ac. It is a cell-permeable sialic acid analog that is converted by CMAS into the corresponding analog of the sialyltransferase donor substrate CMP-3F_ax_Neu5Ac, which is a transition state inhibitor of sialyltransferases (61). Systemic administration in mice reduces sialylation in many tissues, but over several months, results in liver toxicity and kidney dysfunction (62). To explore its utility for stimulating antitumor responses, Büll *et al.* showed that intratumoral injection of 3F_ax_Neu5Ac in melanoma and neuroblastoma tumors resulted in remarkable enhancement of immune responses mediated by CD8+ T cells (55). Targeted delivery to melanoma cells was also achieved by encapsulating 3F_ax_Neu5Ac in poly(lactic-co-glycolic acid) (PLGA) nanoparticles decorated with anti-Trp-1 antibodies (56). In a murine lung metastasis model, intravenous administration of the targeted nanoparticles resulted in a significant reduction in metastatic tumor formation (56). Combination treatment regimens of 3F_ax_Neu5Ac with other inhibitors have also been investigated for enhanced efficacy (63). N-carbamate derivatives of 3F_ax_Neu5Ac have also been developed to enhance potency and prolong inhibitory activity (64). *In vivo* administration of these inhibitors effectively blocked sialylation and decreased metastasis (65).

Due to concerns about toxicity associated with pan-sialyltransferase inhibition and growing evidence that the upregulation of specific sialyltransferases drives cancer progression, there is increasing interest in developing selective sialyltransferase inhibitors. For example, ST6Gal1 upregulation in prostate cancer tissues is correlated with aggressive tumor progression and worse patient outcomes (66). A lithocholic acid derivative, FCW393, was developed to preferentially inhibit ST6Gal1 (IC_50_ = 7.8 μM) and ST3Gal3 (IC_50_ = 9.45 μM) over ST3Gal1 (IC_50_ > 400 μM) and ST8Sia4 (IC_50_ > 100 μM) (67). In murine breast cancer and melanoma models, treatment with FCW393 reduced tumor growth and metastasis, indicating the therapeutic potential of small-molecule sialyltransferase inhibitors (67). Other chemical approaches to develop sialyltransferase inhibitors have been reviewed in detail elsewhere (68). While the development of selective sialyltransferase inhibitors is still in its early stages, ongoing research holds promise for creating targeted therapies that address aberrant glycosylation in cancer without the drawbacks of non-specific inhibition (14).

### Targeting sialidase to tumors

Enzymatic approaches to reduce hypersialylation in the TME have emerged as a promising strategy to enhance antitumor immunity. By selectively removing sialic acid residues from tumor cells, immune cells, or stromal components, these methods aim to remodel the cancer glycocalyx, disrupt glyco-immune checkpoints, and restore immune recognition. Bertozzi and colleagues first pioneered antibody-sialidase conjugates to selectively remove sialic acids from tumor cells. Targeting sialidase to HER2+ cells using trastuzumab effectively desialylated HER2+ cells and improved tumor control *in vivo* (53, 69, 70). In a similar approach, Pedram *et al.* targeted a O-glycoprotease (mucinase) that hydrolyzes mucin proteins densely coated with O-linked sialoglycans overexpressed on tumor cells to the tumor microenvironment instead of a sialidase (71). In addition to targeting tumors, sialidase conjugates have been developed to target immune cells. Using immune checkpoint blocking antibodies, these therapies aim to synergize the effect of immune checkpoint blockade and desialylation in a single treatment (72, 73). Targeted desialylation can be further combined with immune cell engagement to achieve synergistic antitumor effects. For example, Yang *et al.* developed a bispecific T cell engager (BiTE)-sialidase that significantly improved tumor control by strengthening the immune synapse formation between T cells and tumor cells (74, 75). Enzymatic desialylation has recently progressed to clinical trials in the GLIMMER-01 trial that is evaluating E−602, a bi-sialidase Fc fusion protein, in patients with advanced cancers. This ongoing multi-center phase I/II trial will test the safety, tolerability, pharmacokinetics, and antitumor activity of E−602 alone or in combination with anti-PD-1 antibody cemiplimab (NCT05259696) (76).

## Therapies targeting sialic acid receptors

### Therapies targeting the Siglec-sialic acid axis

Siglecs have gained increasing interest as targets in cancer, both for the delivery of drugs to cells that express them, and for their roles as immune checkpoints that suppress antitumor immunity through interactions with tumor-associated sialoglycans. Their relatively restricted expression pattern has led to their use as targets in hematologic malignancies, while their ability to recognize hypersialylation on cancer cells has led to diverse therapeutic strategies that aim to block their interactions or harness them to disrupt tumor sialylation (Table 1). This section will be divided into two key therapeutic approaches: (i) Siglec-targeted drug delivery—approaches that exploit Siglec expression to selectively deliver drugs, and (ii) Targeting Siglecs as immune checkpoints—strategies designed to disrupt Siglec-sialoglycan interactions.

**Table 1:** Therapy modalities aimed at the sialic acid-Siglec axis

Siglec family member	Antibody (Ab)	Antibody-drug conjugate (ADC)	CAR-T therapy	Degrader	Bispecific cell engager	Liposomal or nanoparticle formulation
Siglec-1 (CD169)	No	No	No	No	No	Preclinical (131, 132, 133, 134, 135, 136)
Siglec-2 (CD22)	Yes (78, 79, 80, 81, 82, 83)	Yes (86, 232)	Yes (92, 93, 94, 95, 96, 97, 98, 233)	No	Yes (99, 100)	Preclinical (87, 88)
Siglec-3 (CD33)	Yes (103, 234, 235, 236, 237, 238)	Yes (104, 105, 106, 107, 108, 109, 239, 240, 241)	Yes (118, 119, 120, 121, 242, 243, 244, 245)	No	Yes (114, 115, 116, 117)	No
Siglec-6	No	No	Preclinical (246)	No	No	No
Siglec-7	Preclinical (148, 149)	No	No	Preclinical (150)	Preclinical (149)	No
Siglec-9	Preclinical (154)	No	Preclinical (156)	Preclinical (150)	No	No
Siglec-10	Preclinical (165, 166)	No	No	Preclinical (247)	No	Preclinical (248)
Siglec-15	Yes (171, 176, 177, 178, 179, 180)	No	No	No	No	No

Rows indicate Siglec family members; columns list therapeutic modalities. “Yes” indicates at least one therapy in that category has been reported; “No” indicates none to date.

### Siglec-targeted drug delivery

Siglecs are promising therapeutic targets due to their restricted expression patterns and immunomodulatory roles. Siglec-specific interactions have been leveraged to develop a variety of strategies for drug delivery and targeted killing, including monoclonal antibodies, antibody-drug conjugates, bispecific cell engagers, and CAR-T cells. CD22 and CD33 have been extensively targeted because of their expression on cancer cells in hematologic malignancies.

*CD22 (Siglec-2)*. CD22 is an inhibitory Siglec expressed mostly on B cells. Because of its restricted expression, CD22 is a target for the treatment of B-cell-related malignancies and numerous therapies have been approved or are in clinical trials (77). One of the first to target CD22 is epratuzumab, an anti-CD22 humanized monoclonal antibody (IgG1k) that induces endocytosis of CD22 upon binding, functioning primarily through immunomodulation of B cell function instead of causing B cell depletion (78). Epratuzumab has been evaluated in clinical trials as a monotherapy or in combination with standard chemotherapy or rituximab for treatment of B cell-related malignancies, including NHL and DLBCL (79, 80, 81, 82, 83). To strengthen the effect of monoclonal antibodies, antibody-drug conjugates were developed to deliver toxins to antigen-expressing cells (84). One of the few FDA-approved antibody-drug conjugates specifically targets CD22 (85). Inotuzumab ozogamicin (Besponsa) is indicated for use in the treatment of adults with relapsed or refractory B-cell precursor acute lymphoblastic leukemia (Table 2) (86). Inotuzumab ozogamicin consists of a CD22-specific humanized monoclonal antibody conjugated to N-acetyl-gamma-calicheamicin dimethylhydrazide. Calicheamicin is released following ADC internalization and induces double-stranded DNA breaks, resulting in apoptosis (86). Therapeutic strategies to selectively deliver cytotoxin doxorubicin to B-ALL cells by encapsulation in liposomes decorated with αCD22 antibodies or high-affinity CD22 ligands have also been explored in preclinical models (87, 88).

**Table 2:** FDA-approved Siglec-targeted therapies

Therapy name	Target	Indication	Mechanism	FDA approval status
Inotuzumab Ozogamicin (Besponsa)	CD22 (Siglec-2)	Relapsed or refractory CD22+ B-cell precursor acute lymphoblastic leukemia (ALL)	An anti-CD22 monoclonal antibody conjugated to calicheamicin	Approved for adult patients and pediatric patients ≥ 1 year
Gemtuzumab Ozogamicin (Mylotarg)	CD33 (Siglec-3)	Relapsed or refractory CD33+ acute myeloid leukemia (AML)	An anti-CD33 monoclonal antibody conjugated to calicheamicin	Initially approved in 2000, withdrawn in 2010, re-approved in 2017 with modified dosing regimens for adults and pediatric patients ≥ 2 years
Moxetumomab Pasudotox (Lumoxiti)	CD22 (Siglec-2)	Relapsed or refractory hairy cell leukemia (HCL) in patients who have received at least two prior systemic therapies, including treatment with a purine nucleoside analog	An anti-CD22 monoclonal antibody fragment fused with cytotoxin *Pseudomonas* exotoxin A.	FDA-approved for adults in 2018; voluntarily withdrawn from the U.S. market in 2022 for commercial reasons but remains FDA-approved

Rows list individual therapies; columns indicate the Siglec target, approved clinical indication, therapeutic mechanism, and current FDA approval status.

CAR-T therapy is an emerging field that has demonstrated profound clinical progress in treatment of hematological malignancies (89). In this treatment modality, patient T cells are genetically modified with a chimeric antigen receptors designed to recognize tumor antigens (90). Specifically, Tisagenlecleucel (Kymriah), Axicabtagene Ciloleucel (Yescarta), Brexucabtagene Autoleucel (Tecartus), and Lisocabtagene Maraleucel (Breyanzi), all CAR-T therapies targeting CD19 on B cells, have demonstrated robust clinical efficacy (91). Despite this advancement, antigen escape presents a considerable challenge for long-term therapeutic benefit. Therefore, CAR-T cells targeting other B cell proteins are of considerable interest (Table 3). Due to its relatively restricted expression on B cells, CD22 presents a promising alternative, or supplemental, target to CD19. Accordingly, bi-specific CAR-T cells that recognize both CD19 and CD22 are currently under investigation in clinical trials (Table 3) (92, 93, 94, 95, 96). Overall, CD22-targeting CAR-T therapies have demonstrated substantial efficacy and have been surveyed in detail elsewhere (97, 98). Other therapies are currently in development to further expand the panel of therapies targeting CD22, including CAR-NK cells and CD22-targeted bispecific T cell engagers (BiTEs) (99, 100, 101).

**Table 3:** Select ongoing phase I/II clinical trials with Siglecs

Siglec	NCT number	Patient population	Treatment intervention	Treatment comparison	Outcome measures	Timing/phase	Setting
CD22	NCT05442515	People aged 3–39 with ALL or related B-cell lymphoma that has not been cured by standard therapy	CD19/CD22-directed CAR T-cell therapy	None (single-arm study)	Primary: Overall response rate, safety Secondary: Duration of response, progression-free survival, feasibility	Phase I/II; Recruiting	Multicenter; USA
CD22	NCT03739814	Older, transplant-ineligible patients with newly diagnosed, (Ph)-, CD22+ B-cell acute lymphoblastic leukemia	Inotuzumab Ozogamicin followed by Blinatumomab	Alternative treatment design	Primary: Tolerability, 1-year event-free survival Secondary: 1-year, 3-years OS, RFS, EFS, CR, ORR, MRD	Phase II; Recruiting	Multi center; USA
CD22	NCT04150497	Relapsed or refractory CD22+ B-cell ALL	CD22-directed CAR T-cell therapy (UCART22)	None (single- arm study)	Primary: Safety and tolerability; Secondary: Overall response rate, duration of response, progression-free survival	Phase I/II; Recruiting	Multi center; International
CD22	NCT01371630	Adult patients with pre-B cell ALL, either newly diagnosed or relapsed or refractory	Inotuzumab ozogamicin + low-intensity chemotherapy	3-arm divided by dosing and previous exposure	Primary: Dosing, efficacy by PFS, CR rate, 1-year survival	Phase I/II; Active, recruiting	Single center; USA
CD22	NCT05607420	Relapsed or refractory B-cell non-Hodgkin lymphoma	CD20/22-directed CAR T-cell therapy (UCART20x22)	None (single-arm study)	Primary: Dosing, safety and tolerability; Secondary: Overall survival, objective response rate, PFS	Phase I/II; Recruiting	Multicenter; International
CD33	NCT01409161	Newly diagnosed acute myeloid leukemia (AML) (Adults)	Gemtuzumab ozogamicin (anti-CD33 ADC) + standard induction chemotherapy	None (single-arm study)	Primary: Event-free survival; Secondary: CR rate	Phase II; Active, recruiting	Multicenter; USA
CD33	NCT05984199	Relapsed or refractory AML	CD33-directed CAR T-cell therapy (VCAR33) after allogeneic hematopoietic cell transplantation	None (single-intervention study)	Primary: Safety Secondary: Incidence of GVHD overall response rate, duration of response, progression-free survival	Phase I/II; Recruiting	Multi center; USA
Siglec-6	NCT05488132	Relapsed or refractory AML	Siglec-6-directed CAR T-cell therapy	None (single-arm study)	Primary: Safety and efficacy; Secondary: ORR, MRD, PFS, EFS, OS	Phase I/II; Unknown	Single center; China

Column descriptions are as follows: NCT Number: Identification assigned to each clinical trial registered on ClinicalTrials.gov. Patient Population: Relevant inclusion or exclusion criteria. Treatment Intervention: Brief description of therapy under investigation. Treatment Comparison: Brief description of comparator (*e.g.*, placebo or standard of care). Outcome Measures: List of primary and secondary endpoints. Timing: Specifies the trial phase and status. Setting: Identifies single- or multi-center trials. Trial details were obtained from clinicaltrials.gov.

*CD33 (Siglec-3)*. The exclusive expression of CD33 on myeloid cells make it a significant target for treatment of acute myeloid leukemia (AML) (102). Indeed, 85 to 90% of pediatric and adult AML cases are CD33+ (103). The very first antibody-drug conjugate, initially approved by the FDA in 2000, was gemtuzumab ozogamicin (Mylotarg) targeting CD33 (Siglec-3) for treatment of myeloid leukemia (Table 2) (104, 105). Mylotarg, comprised of calicheamicin conjugated to a humanized monoclonal anti-CD33 antibody was withdrawn due to lack of benefit in 2010, but eventually returned to market in 2017 with altered dosing (104, 106, 107). In patients with relapsed or refractory AML, Mylotarg was demonstrated to extend event-free survival and improve overall survival (108, 109). In addition to the development of other CD33-targeted immunotoxins, a radioimmunotherapy targeting CD33 was recently designed to enhance the potency of monoclonal antibody lintuzumab (humanized IgG). Lintuzumab conjugated to radioisotope actinium Ac 225, known as SGN-33 AC-225, selectively delivers four alpha particles to CD33+ cells (110). In phase I clinical trials, SGN-33 Ac-225 demonstrated an acceptable safety profile and preliminary indications of efficacy (111, 112).

Although several bispecific T cell-engagers have been developed to target T cells to CD33+ cells, most phase I clinical trials evaluating safety and tolerability were discontinued due to on-target off-tumor toxicity (113). However, the AM564 BiTE construct was tolerated in phase I trials in patients with recurrent/refractory (r/r) AML or intermediate to high-risk myelodysplastic syndrome and showed evidence of clinical benefit (114, 115, 116). This difference in safety and efficacy might be attributed to its bivalent CD33 binding moiety that directs T cell cytoxicity to cells with high CD33 expression. Since CD33 is expressed on normal hematopoietic stem cells and myeloid progenitors, this feature could enhance selectivity toward AML blasts. Interestingly, AM564 was investigated for its ability to deplete MDSCs *via* their expression of CD33 and reductions in circulating MDSC in patients treated with AM564 were observed (117).

In contrast to the clinical success achieved in CAR-T therapy for B-ALL, limited efficacy has been demonstrated for CAR-T therapies targeting any antigen in AML (Table 3). For a detailed perspective of CAR-T therapy for AML treatment, including therapies targeting CD33, please refer to this review (118). Although case reports suggest efficacy of CD33-targeted CARs, phase I/II trials have not yet demonstrated clinical efficacy.^120–123^Accordingly, new strategies are being tested to address the challenges faced in CAR-T therapy for the treatment of AML. For example, a dual-targeting CAR that recognizes both CD123 and CD33 has been developed to address the issue of antigen heterogeneity. In a phase 1 clinical trial, this approach induced complete remission in one patient (119). Split CAR designs that dimerize upon exposure to a drug allow CAR activity to be modulated *in vivo*, a mechanism that could be useful to avoid myelotoxicity as CD33 is expressed on both normal and malignant myeloid cells (118). Appelbaum *et al.* developed a rapamycin-induced dimerizing agent-regulated immunoreceptor complex (DARIC) targeting CD33+ tumors (DARIC33) and found potential anti-tumor impact in a phase I clinical trial (120, 121).

As an alternative to CAR-T cells, CAR-NK cells have been investigated for more favorable safety profiles, limited proliferative capacities, as well as non-CAR-dependent antitumor activity. In preclinical models, CD33-targeted CAR-NK reduced leukemic burden in xenograft models with no observable side effects (122, 123). This advancement led to the development of off-the-shelf CAR-NK cells investigated in phase I trials for patients with r/r AML (124, 125). Although results from phase I trials investigating CD33-CAR-NK in patients with r/r AML demonstrated the safety of this treatment, efficacy was limited by expansion of NK cells, and long-term survival was only achieved in one patient (126).

*Siglec-1 (CD169).* CD169 is predominantly expressed on macrophages in lymph nodes, spleen, and bone marrow, making it a promising target for delivering therapeutic agents and vaccines to the immune system. In tumor immunity, CD169+ macrophages play a dual role—while presence of CD169+ macrophages in lymph nodes is indicative of enhanced survival, increased CD169+ macrophages within the tumor microenvironment (TME) is correlated with reduced survival (127, 128). As stated previously, CD169 has no signaling domain, so its value as a target depends on its efficient endocytic properties to deliver cargo to the cell. Due to the role of CD169+ macrophages in orchestrating anti-tumor responses, several groups have explored methods to deliver antigen to this subset as an immunization strategy. In porcine models, antigen delivery *via* an anti-CD169 antibody induced antigen-specific immune responses (129, 130). Antigen delivery to CD169+ macrophages can also be achieved using liposomal platforms decorated with either synthetic high-affinity or endogenous ganglioside CD169 ligands (131). Several reports demonstrate robust induction of iNKT or T cells responses with ligand-functionalized liposomes encapsulating lipid or peptide antigens, respectively (132, 133, 134, 135, 136).

Alternatively, a similar approach was explored by Park and colleagues to deliver toxin to CD169+ macrophages with the goal of depleting tumor-associated macrophages (137). In a murine tumor model, docetaxel-loaded liposomes decorated with CD169 ligands inhibited tumor progression (138). In another approach to reduce TAM-mediated immunosuppression, Tang *et al.* encapsulated zoledronic acid in CD169-targeted liposomes to remodel M2, or anti-inflammatory, TAMs (137). Zoledronic acid has previously been shown to inhibit M2 polarization (139). This approach reduced tumor progression in a syngeneic tumor model (137). Finally, novel glycoengineering approaches to recruit CD169+ macrophages to the tumor microenvironment have been explored by installing CD169 ligands on tumor cells *in vivo* (140, 141). Accumulation of TCCNeu5Ac in the TME led to preferential metabolic uptake and cell-surface display of the high affinity CD169 ligand on tumor cells, recruiting macrophages to suppress both primary tumor growth and metastasis (141).

### Targeting Siglecs as immune checkpoints

Inhibitory Siglecs play a critical role in immune suppression, mediating negative regulation through a mechanism analogous to the PD-1/PD-L1 immune checkpoint interaction. Upon binding to sialoglycan ligands, inhibitory Siglecs recruit protein tyrosine phosphatases *via* cytoplasmic ITIMs to dampen cellular activation signals, enabling tumor immune evasion. A notable exception is Siglec-15, which functions as an activatory receptor, but can still be targeted for therapeutic benefit, as detailed below. Therapeutic strategies aimed at blocking Siglec-mediated suppression have gained traction, particularly in targeting key inhibitory Siglecs in the TME. Approaches include monoclonal antibodies, small molecule inhibitors and degraders.

*Siglec-7.* Targeting Siglec-7 has recently emerged as a promising strategy in cancer immunotherapy. Siglec-7 is an inhibitory receptor expressed on NK cells, monocytes, and macrophages. Wang *et al.* also reported that, within the TME, T cells acquire Siglec-7 and Siglec-9 from interacting monocytes and macrophages through trogocytosis, impairing T cell activation and effector function. These findings underscore the need to consider extrinsically acquired checkpoints on T cells when both developing and applying checkpoint blockade therapies in patients. Siglec-7 promotes tumor immune evasion by suppressing NK cell cytotoxicity, dampening T cell responses, and polarizing TAMs toward an anti-inflammatory phenotype (142, 143, 144, 145, 146, 147). In preclinical models, blocking antibodies have reduced tumor burden and rescued T cell function (142). Ibarlucea-Benitez *et al.* engineered the Fc domain of anti-Siglec-7 antibodies with a point mutation (D265 A) to prevent engagement of FcγRs, and demonstrated this antibody decreased metastatic foci in an *in vivo* model of melanoma (148). In combination with αPD-1treatment, anti-Siglec-7 mAbs can improve tumor control in a synergistic manner (149). The ability of Siglec-7 blockade to enhance the efficacy of αPD-1 therapy in preclinical models suggests its potential utility for treating patients who do not respond to traditional checkpoint inhibitors. Alternatively, the expression of Siglec-7 on NK cells can be exploited to direct NK cell cytotoxic activity (149). Bordoloi *et al.* developed a bi-specific NK cell engager (BiKE) that binds Siglec-7 on NK cells and tumor antigen on tumor cells (149). Treatment with this BiKE improved survival in a murine model of ovarian cancer.

Due to the functional and structural overlap of Siglec-7 and Siglec-9, a dual-targeting degrader was developed to direct Siglec-7 and Siglec-9 to the lysosome for degradation (150). This approach demonstrated robust tumor control in multiple anti-PD-1 refractory preclinical tumor models and synergized with CTLA-4 blockade to reprogram the TME (150). Hong *et al.* also explored a glycoengineering strategy to block Siglec-7 on NK cells by creating high-affinity *cis* ligands *in situ* on the cell surface (151). By preventing Siglec-7-mediated engagement with ligands displayed in *trans* on tumor cells, this strategy enhanced NK cell cytotoxicity *in vitro* (151).

*Siglec-9.* Siglec-9 is an inhibitory receptor expressed on neutrophils, macrophages, and some NK and tumor-infiltrating T cells. Siglec-9 restrains anti-tumor immunity by suppressing neutrophil cytotoxicity, promoting an immunosuppressive phenotype in TAMs, and dampening T cell activation and proliferation (143, 144, 148, 152, 153). Emerging therapeutic strategies to restrict Siglec-9 function include blocking antibodies and bi-specific T cell engagers. A monoclonal antibody generated by immunizing mice with Siglec-9-encoding cDNA and Siglec-9 protein was shown to suppress Siglec-9 signaling *in vitro*, an effect that resulted in reduced tumor progression in an ovarian carcinoma model (SKOV3) (154). Delvaris *et al.* explored an alternative glycoengineering approach to block Siglec-9 interactions by installing synthetic lipid-conjugated glycopolypeptide agonists on the cell surface (155). When tethered on the membrane of immortalized or primary macrophages, Siglec-9 ligands displayed on lipid-linked glycopeptide scaffolds bound to Siglec-9 in *cis*, blocking *trans* interactions and limiting immunosuppressive signaling (155).

The abundance of Siglec-9 ligands on many types of tumor cells has also inspired the development of strategies using the Siglec-9 binding domain to target hypersialylated tumor cells. For example, Eisenberg *et al.* developed a chimeric switch receptor CAR-T cell that uses a construct made up of the extracellular domain of Siglec-9 fused to the intracellular domain of CD28 to convert inhibitory signals into activating ones upon binding to tumor-associated sialic acids (156). CD28 is a costimulatory protein expressed on T cells that amplifies TCR signaling. In a murine xenograft model, Siglec-9 chimeric switch receptor CAR-T cells demonstrated robust tumor control.

In addition, AL009 is an engineered fusion protein designed to act as a “sialic acid trap.” This construct is made up of the extracellular domain of Siglec-9 linked to the Fc domain (157). Promising results from preclinical models show that AL009 can reprogram TAMs into a pro-inflammatory state, reducing primary tumor growth and secondary metastases in a murine model of melanoma either as a monotherapy or in combination with immune checkpoint blockade (157).

*Siglec-10.* Siglec-10 is an inhibitory receptor expressed on various immune cells, including B cells, macrophages, subsets of regulatory T cells and NK cells, with high expression on TAMs. Its expression on macrophages is proposed to suppress anti-tumor immunity through interactions with CD24 on tumor cells, which inhibit phagocytosis, preventing immune clearance (45, 158). Tumors exploit this pathway by upregulating CD24, creating a “don’t eat me” signal that facilitates immune evasion (45, 159). Notably, high expression of Siglec-10 on TAMs correlates with a signature of CD8+ T cell exhaustion, further contributing to immune suppression (160). Xiao *et al.* discovered that TAMs expressing high levels of Siglec-10 produced more suppressive cytokines and inhibitory receptors than TAMs expressing low levels of Siglec-10 (161). Additionally, a subset of regulatory T cells was also shown to express Siglec-10 and interact with soluble CD52 to suppress TCR signaling (162). Elevated Siglec-10 expression in the tumor microenvironment is negatively correlated with patient survival, highlighting its role as a potential therapeutic target (163). Blocking Siglec-10 interactions enhances anti-tumor activity and numerous neutralizing antibodies are currently under investigation (164). Blocking Siglec-10 interactions with recombinant Siglec-10 Fc chimera in a culture of patient HCC tumors processed to a single-cell suspension restored CD8+ T cell-mediated anti-tumor activity, an effect that was synergistic with anti-PD-1 treatment (161). Blocking Siglec-10 in gastric cancer patient tumor cell suspensions also enhanced CD8+ T cell cytokine production *in vitro* and inhibited tumor progression in a humanized mouse (165). A humanized monoclonal antibody AK007 reduced tumor progression in a syngeneic colon adenocarcinoma model in transgenic mice expressing human Siglec-10 (166). It is worth noting that therapies targeting CD24 are in development to block the other side of the interaction (167). Additional information preclinical studies and clinical trials relevant to the CD24/Siglec-10 interaction are covered in a recent review (164).

*Siglec-15.* Siglec-15 is expressed on both immune cells and tumor cells within the tumor microenvironment, making it a significant target in cancer immunotherapy (168, 169, 170, 171). It is an activatory Siglec that interacts with immunoreceptor tyrosine-based activatory motif (ITAM)-bearing adapter proteins through a positively charged amino acid in its transmembrane region (172). For example, Siglec-15 is known to regulate osteoclast differentiation by interacting with DAP12, leading to downstream RANK signaling that results in osteoclast maturation (173). In a breast cancer metastasis model, Wang *et al.* found that Siglec-15 upregulated on macrophages and osteoclasts facilitates the growth of bone cancer and secondary metastases by promoting tumor cell-mediated osteoclastogenesis and inhibiting T cell activity (168). Siglec-15 is reported to suppress T cell activation, proliferation, and cytokine production through *trans* ligation with CD11b on T cells (174). Blocking Siglec-15 with monoclonal antibodies has shown promise in inhibiting tumor growth and bone metastases in murine models (170, 171, 175). One humanized IgG1 mAb NC318 showed promising results in phase I clinical trials and is currently under investigation in a phase II trial that investigates NC318 as a monotherapy or in combination with pembrolizumab, an anti-PD-1 blocking antibody, in patients with locally advanced or metastatic NSCLC (176, 177). Other monoclonal antibodies have shown promising results in preclinical studies or phase I clinical trials (170, 178, 179, 180). To further counteract the immunosuppressive milieu in the TME, Shen *et al.* developed a bispecific antibody targeting both TGF-β, a growth factor associated with tumor invasiveness, and Siglec-15 that suppressed tumor growth in a murine model of triple negative breast cancer that lacks expression of the estrogen receptor, progesterone receptor, and gene for human epidermal growth factor (4T1) compared to mAbs targeting TGF-β and Siglec-15 individually (181).

Other synthetic inhibitors have been explored to block Siglec-15 interactions as well. A small molecule, SHG-8, induced apoptosis in colorectal cancer cells and downregulated Siglec-15 expression *in vitro*. SHG-8 binds near the conserved arginine in Siglec-15 that mediates sialic acid binding, Arg143, with an IC_50_ of 20 μM (182). Wu *et al.* also developed a blocking aptamer that prevented Siglec-15-mediated inhibition of T cell proliferation and delayed tumor progression in a syngeneic murine model of triple-negative breast cancer (183).

### Therapies targeting selectins

Selectins are expressed on endothelial cells of blood vessels (E and P selectins), on platelets (P selectin) or on leukocytes (L selectin) and play a variety of essential roles in leukocyte trafficking based on their interactions with glycan ligands expressed on the cognate cell *in trans*. As described above, glycan ligands for all three selectins are based on the sialoglycan epitopes SLe^x^ and SLe^a^. Because cancer cells also express SLe^x^ and SLe^a^, the selectins are believed to play a significant role in cancer metastasis, the process by which cancer cells spread from the primary tumor to distant organs. Establishment of metastatic foci requires cancer cells to adhere to and extravasate through blood vessel walls, a step in which selectins play a key role (184). Since metastasis is a major cause of cancer mortality, selectins are a promising target to improve patient outcomes (185). Selectins also help establish protective bone marrow niches that confer chemoresistance, and their antagonism has been shown to enhance the efficacy of chemotherapy in preclinical models (186, 187, 188). Therapeutic strategies targeting selectin interactions aim to inhibit tumor cell dissemination and metastasis at distant sites or improve immune cell trafficking.

*P-Selectin*. P-selectin is a cell adhesion molecule expressed on activated platelets and endothelial cells of venules in inflamed tissues that can facilitate interactions between circulating cancer cells and the vasculature. Through P-selectin-mediated interactions between platelets and tumor cells, platelets can protect tumor cells from immune recognition by NK cells to enhance survival in circulation during dissemination (189, 190). Platelet adhesion to tumor cells also induces vascular endothelial growth factor (VEGF) secretion, promoting angiogenesis (191, 192). Indeed, tumor-associated platelets from cancer patients were found to have increased levels of pro-angiogenic factors (*i.e.* PDGF, angiopoietin-1, matrix metalloproteinase-2) (191, 193, 194). A comprehensive description of crosstalk between platelets and tumor cells, has been recently reviewed (195). By helping tumor cells bind to the endothelium, P-selectin also promotes extravasation of tumor cells from the bloodstream into distant tissues (196, 197, 198).

High P-selectin expression in the TME is associated with poorer prognosis and several therapies are in development to interfere with P-selectin interactions (199, 200). Although numerous inhibitors, glycomimetics, and blocking antibodies have been explored for the treatment of cardiovascular and inflammatory diseases, fewer studies demonstrate the efficacy of inhibiting P-selectin for therapeutic benefit in cancer (201). In preclinical murine models of multiple myeloma and glioblastoma, treatment with anti-P-selectin antibodies was demonstrated to decrease tumor volume (199). Crizanlizumab, a humanized mAb that received FDA approval in 2019 for treatment of cardiovascular complications arising from sickle cell disease, is currently being investigated in a phase I/II clinical trial to evaluate efficacy, safety, and tolerability in combination with nivolumab for the treatment of patients with glioblastoma or melanoma with brain metastasis (NCT05909618) (Tables 4 and 5) (202, 203). In addition to blocking antibodies, glycomimetics offer an alternative strategy to modulate P-selectin activity. For example, holothurian glycosaminoglycan, a marine-derived polysaccharide, potently inhibited P-selectin binding to tumor cells *in vitro* and prevented formation of metastatic foci in a murine lung metastasis model (204). Feng *et al.* also reported a novel nanoparticle-encapsulated small molecule inhibitor of P-selectin that exhibited similar tumor control to that achieved with standard chemotherapy doxorubicin in a syngeneic murine tumor model (205).

**Table 4:** FDA-approved Selectin-targeted therapies

Therapy name	Target	Indication	Mechanism	FDA approval status
Crizanlizumab-tmca (Adakveo®)	P-Selectin	Reduction of vaso-occlusive crises in patients with sickle cell disease	Monoclonal antibody that binds to P-selectin on endothelial cells and platelets, inhibiting interactions that lead to vaso-occlusion and inflammation	FDA-approved in November 2019 for adults and pediatric patients ≥ 16 years

Rows list individual therapies; columns indicate the Selectin target, approved clinical indication, therapeutic mechanism, and current FDA approval status.

**Table 5:** Key clinical trials targeting P-selectin in cancer

NCT number	Patient population	Treatment intervention	Treatment comparator	Outcome ceasures	Timing/phase	Setting
NCT05909618	Advanced glioblastoma, metastatic brain melanoma, newly diagnosed MGMT-unmethylated glioblastoma	Crizanlizumab alone or with nivolumab	3-arm divided by patient cohort (Single-intervention study)	Primary: Safety and tolerability Secondary: RR, PFS, OS, DCR	Phase I/II, recruiting	Single-center, Sheba Medical Center, Israel

Column descriptions are as follows: NCT Number: Identification assigned to each clinical trial registered on ClinicalTrials.gov. Patient Population: Relevant inclusion or exclusion criteria. Treatment Intervention: Brief description of therapy under investigation. Treatment Comparison: Brief description of comparator (*e.g.*, placebo or standard of care). Outcome Measures: List of primary and secondary endpoints. Timing: Specifies the trial phase and status. Setting: Identifies single- or multi-center trials. Trial details were obtained from clinicaltrials.gov.

PSGL-1 is a major ligand for P-selectin that is expressed on some leukocytes as well as tumor cells in some hematologic malignancies (206). As previously mentioned, PSGL-1 is a unique ligand because it has sulfated tyrosines adjacent to O-linked glycans containing SLe^x^, that increases its affinity for P-selectin (207). High expression of PSGL-1 on multiple myeloma is reported to facilitate homing and adhesion to cells in the bone marrow microenvironment, which contributes to disease progression and confers resistance to traditional chemotherapies (208, 209). GMI-1070, a pan-selectin antagonist that was also developed to treat complications of sickle cell disease, was used to block this interaction in a murine model of multiple myeloma (210, 211). Treatment with GMI-1070 improved survival and restored sensitivity to bortezomib, a proteasome inhibitor (211). Blocking PSGL-1 with a mAb in a xenograft model of cutaneous T cell lymphoma also reduced tumor progression by promoting tumor cell apoptosis through the MAPK signaling pathway (212).

*E-selectin*. E-selectin can facilitate the metastasis of circulating tumor cells that express SLe^x/a^ through its expression on activated endothelial cells in venules of distant tissues. By facilitating tumor cell adhesion to the vasculature, E-selectin plays a crucial role in the early steps of metastasis (213). Normally, E-selectin is not expressed on vasculature endothelium but is rapidly expressed in response to inflammatory stimuli. Recent reports suggest that upregulation of E-selectin on tumor vasculature promotes tumor cell survival by creating a protective niche (214). Winkler and colleagues also demonstrated that vascular E-selectin conferred resistance to chemotherapy in AML *via* pro-survival NF-kB signaling (186, 215, 216). Higher circulating expression of E-Selectin is also associated with shorter event-free survival and is predictive of relapse in AML patients (217). Therefore, there is considerable interest in developing strategies to interfere with E-selectin interactions.

A potent glycomimetic inhibitor of E-selectin, uprolesalan (GM-1271, K_d_ = 0.54 μM) reduced tumor burden in humanized mice in combination with standard chemotherapy agents (218). Further promising results in preclinical models demonstrated uprolesalan extended survival in a murine model of breast cancer and reduced metastasis in a murine model of pancreatic cancer (219, 220). Phase I clinical trials investigated safety and tolerability in r/r AML patients demonstrated promising results and identified a reduction in rates of oral mucositis, a common side effect of chemotherapy (221, 222). Despite this strong rationale, a phase III trial testing combination treatment of uprolesalan with standard chemotherapy was terminated after failing to meet its primary endpoint (NCT03616470). Several clinical trials evaluating uprolesalan are continuing (Table 6) (223, 224).

**Table 6:** Key clinical trials targeting E-selectin in cancer

Trial ID	Patient population	Treatment intervention	Treatment comparator	Outcome measures	Timing/phase	Setting
NCT02306291	Adults with relapsed/refractory AML or newly diagnosed AML	Uproleselan + MEC chemotherapy or 7 + 3 chemotherapy	None (Single-arm study)	Remission rate (CR/CRi), OS, safety	Phase I/II completed	Multicenter, international
NCT03616470	Adult patients with relapsed/refractory AML	Uproleselan + chemotherapy	Placebo + same chemotherapy regimens	OS (primary), OR rate, mucositis incidence	Phase III; terminated; failed to meet primary endpoint	Multicenter, international
NCT03701308	Adults ≥60 years with newly diagnosed AML	Uproleselan + chemotherapy	Standard chemotherapy	EFS (primary), OS, CR rate, safety	Phase II/III; ongoing	Multi-center
NCT05569512	Pediatric patients with AML undergoing HSCT	Uproleselan in pre-transplant conditioning	Standard pre-transplant conditioning	Dosage, DLT, PK, EFS, OS	Phase I/II; terminated due to company restructuring	Multi-center
NCT04848974	Patients with treated secondary AML	Uproleselan + cladribine + low-dose cytarabine	None (Single-arm study)	Safety, tolerability, dosage, ORR, CRR, MRD	Phase I/II; completed	Single-center, MD Anderson, Houston, TX
NCT05146739	Pediatric patients with relapsed/refractory AML	Uproleselan + fludarabine + high-dose cytarabine	None (Single-arm study)	MTD, PK, safety	Phase I; active	Multi-center

Column descriptions are as follows: NCT Number: Identification assigned to each clinical trial registered on ClinicalTrials.gov. Patient Population: Relevant inclusion or exclusion criteria. Treatment Intervention: Brief description of therapy under investigation. Treatment Comparison: Brief description of comparator (*e.g.*, placebo or standard of care). Outcome Measures: List of primary and secondary endpoints. Timing: Specifies the trial phase and status. Setting: Identifies single- or multi-center trials. Trial details were obtained from clinicaltrials.gov.

Because E-selectin is highly expressed in tumor vasculature and absent on resting endothelium, it is an attractive target for selective delivery of drugs to the tumors (225). Shamay *et al.* targeted the delivery of a chemotherapy drug, doxorubicin, or a proapoptotic peptide D(KLAKLAK)2 through conjugation with an E-selectin-binding peptide, an approach that reduced primary tumor growth and extended survival in a murine lung carcinoma model (226). Treatment with E-selectin-binding peptide alone reduced formation of metastatic foci in a metastatic murine model of melanoma, suggesting it was able to interfere with E-selectin-mediated tumor cell migration (226). A similar approach using doxorubicin-loaded nanoparticles decorated with E-selectin ligands resulted in delayed tumor progression in a murine breast cancer model (227).

E-selectin has also been considered in the context of the recruitment of immune cells to the tumor microenvironment by mediating the adhesion and extravasation of leukocytes. Glycoengineering strategies have therefore been developed to enhance immune cell trafficking to tumor sites. By modifying the surface glycans of immune cells, these approaches aim to increase their homing capabilities and tumor infiltration. Enzymatic fucosylation of NK cells, achieved by *in vitro* incubation with fucosyltransferase and its donor substrate GDP-fucose, resulted in increased cell-surface expression of E-selectin ligands that promoted migration to the bone marrow for enhanced tumor control in murine lymphoma models (228, 229). A similar result was achieved with the *in situ* fucosylation of CAR-T cells to achieve superior tumor control (230, 231).

### Outlook

Targeting the sialic acid axis for treatment of cancer is gaining increasing attention, both for enhancing antitumor immune responses and for selective targeting of therapeutic agents to cancer or immune cells. Clinical success achieved by targeting Siglecs expressed on hematopoietic cancers has motivated efforts to expand the strategy to novel immunotherapy modalities, while blocking Siglec-mediated immune evasion by disrupting interactions with inhibitory sialic acid ligands also shows promise in preclinical studies. Renewed interest in Selectin inhibition to prevent metastasis has led to preclinical success as well, with several therapies under investigation in clinical trials. Despite these advances, the paucity of glycan-targeted therapies in the clinic underscores the need for a deeper understanding of the mechanistic roles of sialoglycans in cancer.

Successful therapies targeting the sialic acid/sialoglycan receptor axis in cancer will require deeper mechanistic insight into these interactions in different contexts. Certain sialic acid receptors, such as Siglecs expressed on suppressive myeloid cells, remain underexplored in cancer, despite clear relevance to tumor progression. For example, CD33 is known to be expressed on a number of cells, but its precise function is not known on any cell. Notably, therapies targeting sialic acid receptors have shown efficacy in other disease areas, such as inflammatory conditions. These advances provide a compelling foundation for repurposing existing therapeutic platforms in the treatment of cancer. Still, specificity of glycan-targeted therapies remains a significant challenge since therapies must selectively target aberrant glycosylation without disturbing the normal glycocalyx to prevent on-target off-tumor toxicity. Taken together, the emerging therapeutic strategies discussed in this review demonstrate the growing potential of sialic acid-targeted approaches in cancer treatment.

## Conflict of interest

The authors declare that they do not have any conflicts of interest with the content of this article.
